# Risk Factors for Foot‐Related Hospitalisations in Adults With and Without Diabetes: A Systematic Review

**DOI:** 10.1002/jfa2.70164

**Published:** 2026-06-08

**Authors:** Sucharitha R. Weerasuriya, Chanika Alahakoon, Nimantha Karunathilaka, Yuqi Zhang, Susanna M. Cramb, Peter A. Lazzarini

**Affiliations:** ^1^ Australian Centre for Health Services Innovation and Centre for Healthcare Transformation School of Public Health and Social Work Queensland University of Technology Brisbane Queensland Australia; ^2^ College of Medicine and Dentistry James Cook University Cairns Campus Cairns Queensland Australia; ^3^ Queensland Research Centre for Peripheral Vascular Disease James Cook University Townsville Queensland Australia; ^4^ School of Nursing Faculty of Health Queensland University of Technology Brisbane Queensland Australia; ^5^ Department of Nursing and Midwifery Faculty of Allied Health Sciences General Sir John Kotelawala Defence University Ratmalana Sri Lanka; ^6^ Department of Medical Epidemiology and Biostatistics Karolinska Institute Solna Sweden; ^7^ Jamieson Trauma Institute Metro North Health Royal Brisbane and Women's Hospital Herston Queensland Australia; ^8^ Allied Health Research Collaborative The Prince Charles Hospital Brisbane Queensland Australia

**Keywords:** diabetes, diabetic foot, foot disease, hospitalisation, risk factor

## Abstract

**Introduction:**

Foot‐related conditions are a leading cause of all hospitalisations and amputations worldwide. Half of these foot‐related hospitalisations are in people without diabetes. Yet, few studies seem to have explored risk factors for foot‐related hospitalisations in populations with or without diabetes. This study aimed to systematically review studies investigating risk factors for hospitalisations caused by any foot‐related conditions amongst any general community‐dwelling adult populations (with or without diabetes).

**Methods:**

PubMed and Embase databases were searched for studies related to risk factors, foot‐related conditions and hospitalisations published since 1st January 2000. Search results were screened for eligibility by two independent authors. Risk of bias was assessed using the Quality in Prognostic Studies tool, and data were extracted using a customised data extraction tool.

**Results:**

Fourteen studies from 7824 screened studies were included. Twelve studies investigated diabetes populations and two general (with and without diabetes) populations. All 14 studies investigated only for foot disease‐related hospitalisation outcomes. Seven studies were rated as low risk of bias. Twenty‐two independent risk factors were reported, including eight reported both in multiple studies and low risk of bias studies. Those eight risk factors were being male, having diabetes, increased HbA1c, insulin management, chronic kidney disease, peripheral neuropathy, peripheral artery disease and no footcare within 12 months.

**Conclusions:**

This review suggests that the common risk factors for foot disease‐related hospitalisations are being male, having diabetes, chronic kidney disease, peripheral neuropathy, peripheral artery disease and lack of footcare, particularly in diabetes populations. There were no studies investigating hospitalisations for other foot‐related conditions and few in nondiabetes populations.

## 1 Introduction

Foot‐related conditions cause around 10% of all hospitalisations [1, 2, 3, 4] and are a leading contributor to the global burden of diseases [5, 6, 7]. Foot‐related conditions are defined as any disorder or condition that affects the foot or the ankle [2, 8, 9]. They are further categorised into foot disease (such as infective, ulcerative/chronic wound, ischaemic/peripheral artery disease (PAD), and neuropathic disorders) and musculoskeletal (such as bone, joint, and soft tissue disorders), dermatological (such as skin and nail disorders) and traumatic (such as acute wounds and injuries) conditions [2, 8, 9]. The conditions categorised as foot disease have been reported to account for around two thirds of all hospitalisations caused by foot‐related conditions (‘foot‐related hospitalisations’) [1, 2], while about half of all foot‐related hospitalisations have been reported to be in people with diabetes [2, 10, 11].

Despite the larger proportion of people hospitalised for foot‐related conditions, to our knowledge, few studies and no reviews have explored the risk factors for being admitted to hospital for foot‐related conditions amongst general (those with or without diabetes) populations living in the community (‘community‐dwelling’). Better understanding the key risk factors for foot‐related hospitalisations is critical to informing practice and policy to potentially prevent many of these hospitalisations in the future. Therefore, this paper aimed to systematically review studies investigating risk factors for hospitalisations caused by any foot‐related condition (including foot disease, musculoskeletal, dermatological or traumatic conditions) among any general community‐dwelling adult populations.

## 2 Materials and Methods

We performed this systematic review in accordance with the Preferred Reporting Items for Systematic Reviews and Meta‐analysis (PRISMA) statement [12] and prospectively registered this review in the PROSPERO international prospective register of systematic reviews database (CRD42024528822).

### 2.1 Search Strategy

We conducted a search on 31st October 2024 of the PubMed (MEDLINE) and Embase databases for relevant studies published from 1st January 2000. We selected these two databases as they have previously been reported to identify all peer‐reviewed studies related to the foot‐related conditions field according to the International Working Group on the Diabetic Foot [13, 14, 15, 16]. We used search strings combining MeSH terms, appropriate synonyms and truncation settings for the terms ‘foot’, ‘conditions’, ‘risk factors’ and ‘hospitalisation’. We finalised our search strategy after ensuring that the strategy identified all 21 studies contained in a validation set that we created of studies considered relevant to this review (Supporting Information S1: Table S1). The search was restricted to the English language, human studies and those studies published since 1st January 2000. We selected this start date as it followed the 1999 publication of the first‐ever international foot disease guidelines by the International Working Group on the Diabetic Foot (IWGDF) [17], which is likely to have substantially changed international foot care practices. All final search strings are shown in Supporting Information S1: Table S2.

### 2.2 Eligibility Criteria

In summary, to be eligible for inclusion in this review, studies had to be of an original observational study design that investigated the association between any exposure variable and an outcome of hospitalisation for a foot‐related condition (‘foot‐related hospitalisation’) in any adult population living in the community (‘community‐dwelling adults’) and not residing in an institution (such as hospital inpatient or aged care facility residents). Populations of interest for this review were any community‐dwelling adult population, including general resident populations or populations with specific comorbidities, such as diabetes, cardiovascular disease or chronic kidney disease populations. Exposures of interest were any variable, such as sociodemographic, biomedical measures, comorbidity, foot‐related condition or management and service‐related variables. Outcomes of interest were any foot‐related hospitalisations. Foot‐related hospitalisation was defined as an admission of a person into hospital overnight for the primary reason (principal diagnosis) of treatment for a foot‐related condition [9]. Exclusion criteria included any populations that were institutionalised (such as in residential aged‐care homes, prisons, hospital inpatients), foot‐related hospitalisations where a foot‐related condition was not the principal cause of admission, and grey literature.

### 2.3 Eligibility Assessment

All records identified from the search were imported into EndNote Version 20 software [18]. Any duplicate records were detected by EndNote, manually checked by the first author and removed. All remaining unique records were then imported into the online application Rayyan to assist with eligibility assessment procedures [19]. Two independent authors (SRW and NK) screened the title and abstracts of all identified unique records using the above inclusion and exclusion criteria. Disagreements were resolved by discussion; where consensus was not possible, a third author decided (PAL or SMC).

Two authors (SRW and CA) then independently assessed the full text of all records determined to be potentially eligible for inclusion after the screening process using the same inclusion and exclusion criteria. Any disagreements were again resolved by discussion or a third author decided (PAL or SMC). If an author had co‐authored a record, they were excluded from that record's assessment. We included all full texts deemed fully eligible after this process as the final included studies in the systematic review, and reasons for inclusion or exclusion of full texts were documented.

### 2.4 Risk of Bias Assessment

Two authors (SRW and CA) then independently assessed the risk of bias (RoB) of all included studies using the revised version of the validated Quality in Prognostic Studies (QUIPS) tool [20]. The QUIPS tool has been validated [20, 21] and is recommended by the Cochrane Prognosis Method Group for RoB assessments of studies investigating prognostic factors [22]. The QUIPS tool assesses 32 individual items across six different RoB domains: participation, attrition, prognostic factor/exposure measurement, outcome measurement, confounders, and statistical analysis and reporting. Specific criteria to meet each item for the purposes of this systematic review were developed, discussed and agreed by consensus of three authors (SRW, SMC and PAL) as per QUIPS recommendations and are listed in Supporting Information S1: Table S3. We assessed the statistical analysis and reporting domain using the Statistical Analyses and Methods in the Published Literature (SAMPL) guideline for biomedical journals [23], and the full criteria are listed in Supporting Information S1: Table S4. Each domain's RoB was assessed based on the collective assessments of the individual items in that domain as high, moderate or low RoB. Domains were rated as low RoB, if all domain items were assessed as ‘yes’ or if only one item was assessed as ‘partial’; high RoB, if two or more domain items were assessed as ‘no’ or ‘unsure’; or moderate RoB, for all other domain item assessment combinations that were not defined as low or high RoB. The final overall RoB assessment of the included study was then assessed based on the collective assessments of the individual domains. Studies were rated as low RoB, if all domains were assessed as low RoB or if only one was assessed as moderate RoB; high ROB, if one or more domains were assessed as high RoB or three or more domains were assessed as moderate; or moderate RoB, for all other domain assessment combinations that were not defined as low or high RoB [20]. Any disagreements on items, domains or overall assessments between authors (SRW and CA) were discussed until consensus was reached, and if this was not possible, a third author (PAL) was asked to decide.

### 2.5 Data Extraction

Two authors (SRW and CA) also independently extracted applicable data from the first 30% of included studies using a custom designed data extraction spreadsheet tool to capture study characteristics, including study reference (first author and year of publication), study setting, population source, study design, study period, population characteristics, outcome reported, variables explored and independent risk factor findings. Any disagreements between authors were discussed until consensus was reached, and if this was not possible, a third author (PAL) was asked to decide. After completion and agreement on the data extraction for the first 30% of included studies, the first author extracted data from the remaining 70% of included studies. If the same participant numbers and variables explored were reported from the same study in two or more publications, we used the findings from the earlier study. However, if different participant numbers and variables explored were reported from the same study, we treated the publications as separate studies.

### 2.6 Data Analysis

The summary measures used for the association between each variable and outcome in each included study were either an adjusted hazards ratio, adjusted odds ratio or adjusted incidence rate. Note studies measuring a similar variable, according to the definitions used in those studies, were grouped together for the ease of reporting. Risk factors were defined as only those variables included in a multivariable analysis or adjusted for other variables (such as age adjusted) and remaining independently and statistically significant according to the confidence interval (not crossing 1) or *p*‐value (*p* < 0.05) reported. Eligibility for performing a meta‐analysis required at least three studies reporting the association between the same risk factor and the same outcome in the same population of interest using similar definitions. In this case, a meta‐analysis would be used to calculate the pooled effect measures of the risk factor on the outcome of interest.

## 3 Results

### 3.1 Search Results

**FIGURE 1:**
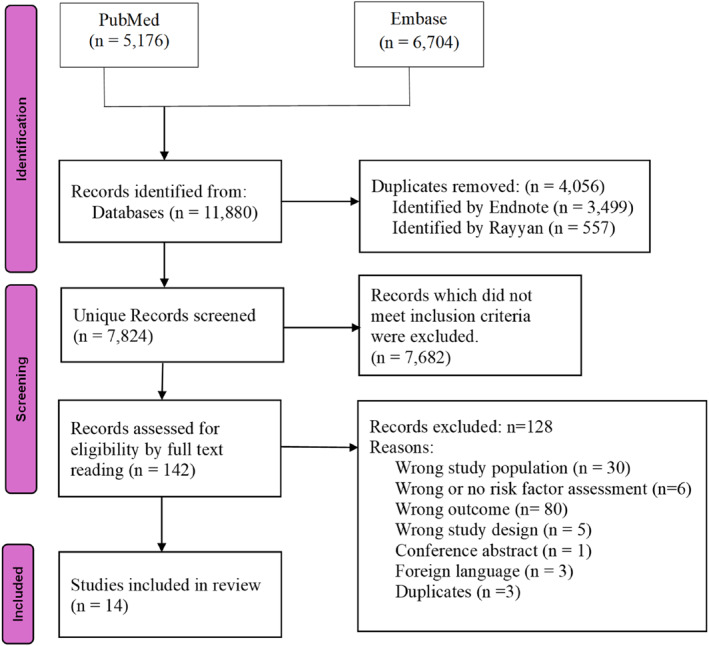
Preferred Reporting Items for Systematic Reviews and Meta‐Analyses (PRISMA) flow diagram.

A total of 11,880 studies were identified from the search strategy. After removal of 4056 duplicates, 7824 unique studies remained. After title and abstract screening, 142 remained. After full text assessment, 14 studies met eligibility criteria and were the final included studies in this review (see PRISMA flow diagram in Figure 1). The eligibility criteria for performing a meta‐analysis were not met for any risk factor, and therefore, only qualitative analyses of included studies are reported.

### 3.2 Data Extraction

Table 1 displays the summary study characteristics for the 14 included studies, including nine population‐based studies, two multicentre studies and three single‐centre studies. The studies were conducted in seven countries: four in Australia [24, 25, 28, 34], four in USA [26, 27, 35], two in Italy [32, 33] and one each in Canada [31], France [29], Korea [30] and Peru [37].

**TABLE 1:** Summary study characteristics of included studies.

Reference	Study setting	Population source	Study design	Study period	Definition	Population characteristics			Outcome definition	Independent risk factors reported	Risk of bias (RoB) status
						Numbers *(n)*	Age years mean (SD)	Male sex %			
Baba et al. 2014 [24]^b^	Fremantle, Australia	Population‐based	Prospective cohort study	1996–2013	Type 2 diabetes	*n* = 1292	64 (11.0)	48.6%	Foot ulcer–related hospitalisation identified from relevant foot ulcer ICD hospital codes and verified in medical records	Alcohol consumption, HbA1c level, retinopathy, cerebrovascular disease, chronic kidney disease (eGFR < 60), peripheral neuropathy, PAD, intermittent claudication and pulse pressure	High
Dinh et al. 2022 [25]	Tasmania, Australia	Population‐based	Retrospective cohort study	2007–2017	General residents	*n* = 136,134	62 (15.0)	55.0%	Foot ulcer‐ or gangrene‐related hospitalisation identified from relevant foot ulcer or gangrene ICD hospital codes and verified in medical records	Diabetes	Low
Fang et al. 2021 [26]	North Carolina, Mississipi, Minnesota and Maryland, USA	Population‐based	Prospective cohort study	1987–2019	General residents	*n* = 12,379	55 (6.0)	45.7%	Foot infection related hospitalisation identified from relevant foot infection ICD hospital codes	Diabetes	Low
Gibson et al. 2013 [27]	Nationwide USA	Population‐based	Retrospective cohort study	2005–2010	Diabetes	*n* = 47,753	NR	NR	Foot ulcer related hospitalisation identified from claimed data on stay in an inpatient facility including one night of room and board.	No podiatrist visit	High
Hamilton et al. 2021 [28]^b^	Fremantle, Australia	Population‐based	Prospective cohort study	1996–2016	Type 2 diabetes	*n* = 2741	64.8 (11.5)	50.3%	Foot ulcer related hospitalisation identified from relevant foot ulcer ICD hospital codes.	Height, HbA1c level, age at diabetes diagnosis, peripheral neuropathy, peripheral vascularisation (previous), foot pulse absence and insulin therapy	Low
Ha Van et al. 2023 [29]	Paris, France	Multicentre	Retrospective cohort study	2019–2020	Diabetes	*n* = 537	69.0 (12.4)	75.8%	Foot complication–related hospitalisation: Ulcers complicated with wet gangrene, abscess, fever, signs and sepsis, critical limb ischaemia and need for revascularisation, septic surgery of soft tissue and/or bone or parenteral antibiotic therapy–related hospitalisation	Foot infection, ischaemia	High
Kwon et al. 2024 [30]	Seoul, Korea	Multicentre	Retrospective cohort study	2001–2016	Type 2 diabetes visited nephrology department more than twice	*n* = 10,832	58.9 (12.6)	58.6%	Foot ulcer related hospitalisation identified from relevant foot ulcer ICD hospital codes	Metformin usage	Low
Manji et al. 2024 [31]	Alberta, Canada	Population‐based	Retrospective comparison study	2007–2017	Diabetes	*n* = 312,332	NR	NR	DFD‐related hospitalisation:	Multidisciplinary foot care	High
Meloni et al. 2021 [32]	Rome and Lazio, Italy	Single‐centre	Retrospective cohort study	2019–2020	Diabetes	*n* = 200	70 (13.0)	62.5%	DFU‐related hospitalisation	Ischaemic and late referral to specialised foot services	High
Monge et al. 2019 [33]	Piemont, Italy	Population‐based	Retrospective cohort study	2012–2016	Diabetes	*n* = 4350	NR	61.3%	DFD‐related hospitalisation Identified from relevant DFD‐related ICD hospital codes	Male sex, low education status, cardiovascular disease and previous dialysis	Moderate
Quigley et al. 2022 [34]	New South Wales, Victoria, Queensland and Australian Capital Territory, Australia	Population‐based	Retrospective cohort study	2010–2019^a^	Diabetes	*n* = 965,896	NR	54.4%	DFD‐related diabetes and foot disease–related hospitalisation identified from relevant DFD‐related ICD hospital codes’ hospitalisation	Type 1 diabetes	Low
Schmidt et al. 2023 [35]	Michigan, USA	Single‐centre	Retrospective cohort study	2019–2020	Diabetes with DFI	*n* = 116	58.0 (11.0)	76.7%	Foot infection–related hospitalisation:	Not performing a multivariable analysis	High
Tan et al. 2024 [36]	Los Angeles, USA	Population‐based	Retrospective cohort study	2010–2019	Type 2 diabetes	*n* = 307,131	NR	50.3%	DFU‐related hospitalisation	Preulcerative foot care	Low
Yovera et al. 2024 [37]	Lima, Peru	Single‐centre	Retrospective cohort study	2017–2019	Diabetes with DFI	*n* = 192	59.9 (12.9)	74.0%	DFI‐related hospitalisation	Not identified an independent risk factor	Low

Abbreviations: DFD, diabetes foot disease; DFI, diabetes foot infection; DFU, diabetes foot ulcer; eGFR, estimated glomerular filtration rate; HbA1c, glycated haemoglobin; ICD, International Classification of Diseases; NR, not reported; PAD, peripheral artery disease; SD, standard deviation.

^a^ Numbers reported for 2018–2019.

^b^ Population from the same study; however, Hamilton et al. had many more participants and explore different risk factors for hospitalisation.

A total of 1,799,155 participants were reported across the 14 studies, including 1,782,743 participants in 11 retrospective cohort studies [25, 27, 29, 30, 31, 32, 33, 34, 35, 36, 37] and 16,412 in three prospective cohort studies [24, 26, 28]. In the 11 retrospective studies, 1,770,866 participants were from six population‐based studies [25, 27, 31, 33, 34, 36], 11,379 from two multicentre studies [29, 30] and 508 from three single‐centre studies [32, 35, 37]. In the three prospective cohort studies, all 16,412 participants were from three population‐based studies [24, 26, 28]. The population sources included general populations (diabetes and nondiabetes) in two studies [25, 26], diabetes populations (type 1 and type 2) in eight studies [27, 29, 31, 32, 33, 34, 35, 37] and type 2 diabetes populations only in four studies [24, 28, 30, 36]. The mean age of the participants across the 14 studies ranged from 55 [26] to 70 [32] years, and the proportion of male sex from 46% [26] to 77% [35].

The foot‐related condition causing the foot‐related hospitalisation outcomes of interest was foot disease in all 14 studies, including specifically foot ulcers in eight studies [24, 25, 27, 28, 29, 30, 32, 36], foot infections in three studies [26, 35, 37] and a combination of different foot disease conditions in three studies [31, 33, 34]. A total of 97 variables were explored across the 14 studies, including 15 sociodemographic, 17 biomedical, 19 comorbidity, 30 foot‐related condition and 16 management variables as displayed in Supporting Information S1: Table S5. In terms of reporting independent risk factors, five studies reported adjusted hazards ratios (HRs) [24, 26, 27, 28, 30], five adjusted odds ratios (ORs) [29, 32, 35, 36, 37] and four adjusted incidence rates [25, 31, 33, 34].

### 3.3 Risk of Bias Assessment

The risk of bias (RoB) assessment is shown in Table 2. The overall RoB ratings for the 14 studies included seven (50%) rated as low RoB [25, 26, 28, 30, 34, 36, 37], one (7%) moderate RoB [33] and six (43%) high RoB [24, 27, 29, 31, 32, 35]. Studies recording low RoB ratings for each domain included 13 (93%) studies for outcome measurement, 10 (71%) for study attrition, 9 (64%) studies for prognostic factor measures, 9 (64%) studies for study participation, 8 (57%) studies for study confounding details and one (7%) for statistical analysis and reporting.

**TABLE 2:** Risk of bias scores of included studies.

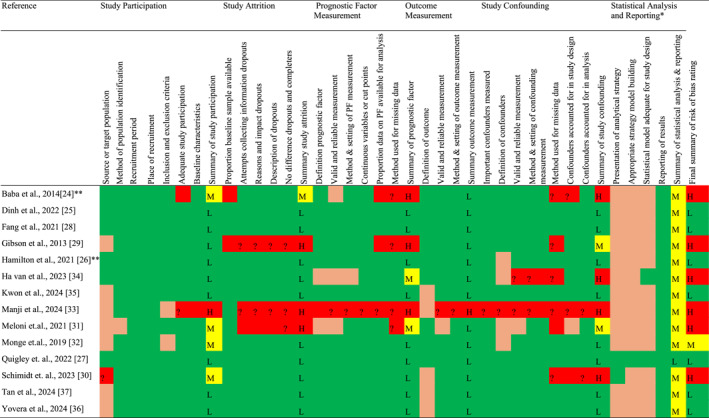

*Note:* Subdomain rating: yes (green), partial (orange), no (red) and unsure (?red). Summary domain rating and overall summary RoB rating: L = low (green), M = moderate (yellow) and H = high (red).

^a^ Statistical Analyses and Methods in the Published Literature (SAMPL) guidelines were used to further define this domain.

^b^ Population from the same study; however Hamilton et al. had many more participants and explored different risk factors for hospitalisation.

### 3.4 Risk Factors Identified

Table 3 displays a summary of the 22 independent risk factors reported for foot‐related hospitalisations across the 14 included studies, including four sociodemographic, one biomedical, eight comorbidity, three foot‐related condition and six management variables. Supporting Information S1: Table S6 displays all risk factor findings and Supporting Information S1: Table S7 reports the definitions used for these factors in included studies. The four sociodemographic risk factors reported were male sex in two studies [33, 34], and lower education status [33], increased height [28], and increased alcohol consumption [24] in one study each. The biomedical risk factor reported was increased HbA1c in two studies [24, 28]. The eight comorbidity risk factors reported were diabetes [25, 26] and chronic kidney disease in two studies each [24, 28], plus type 1 diabetes [34], younger age at diabetes diagnosis [28], cardiovascular disease [33], cerebrovascular disease [24], end‐stage renal failure [33] and retinopathy [24] in one study each. The three foot‐related risk factors were PAD reported in four studies [24, 28, 29, 32], peripheral neuropathy in two studies [24, 28] and foot infection in one study [29]. Finally, the six management risk factors were no professional footcare within 12 months of foot ulcer diagnosis [27, 36] and insulin management [28, 33] in two studies, and oral antidiabetes drugs use [33], metformin nonuse [30], late referral to specialised diabetic foot services [32] and no multidisciplinary limb preservation program [31] in one study each.

**TABLE 3:** Summary of risk factor findings for foot‐related hospitalisations in included studies.

Variables	Reference													
	Dinh et al. 2022 [25]	Fang et al. 2021 [26]	Gibson et al. 2013 [27]	Manji et al. 2024 [31]	Monge et al. 2019 [33]	Quigley et al. 2022 [34]	Ha Van et al. 2023 [29]	Meloni et al. 2021	Schimidt et al. 2023^a^ [35]	Yovera et al. 2024 [37]	Baba et al. 2014^b^ [24]	Hamilton et al. 2021^b^ [28]	Tan et al. 2024 [36]	Kwon et al. 2024 [30]
Population of interest	General		Diabetes								Type 2 diabetes			Type 2 diabetes with kidney disease
Risk of bias status	L	L	H	H	M	L	H	H	H	L	H	L	L	L
Sociodemographic variables														
Males	NR	NR	NR	NR	SIG	SIG	NR	NR	NR	NR	NR	NR	NR	NR
Low education status	NR	NR	NR	NR	SIG	NR	NR	NR	NR	NR	NR	NR	NR	NR
Increased height (1 cm increase)	NR	NR	NR	NR	NR	NR	NR	NR	NR	NR	NR	SIG	NR	NR
Alcohol consumption (1 standard drink/day)	NR	NR	NR	NR	NR	NR	NR	NR	NR	NR	SIG	NR	NR	NR
Biomedical measures														
HbA1c	NR	NR	NR	NR	NR	NR	NR	NR	NR	NR	SIG	SIG	NR	NR
Comorbidities														
Diabetes	SIG	SIG	NR	NR	NR	NR	NR	NR	NR	NR	NR	NR	NR	NR
Type 1 diabetes	NR	NR	NR	NR	NS	SIG	NR	NR	NR	NR	NR	NR	NR	NR
Young age at diabetes diagnosis	NR	NR	NR	NR	NR	NR	NR	NR	NR	NR	NR	SIG	NR	NR
Cardiovascular disease	NR	NR	NR	NR	SIG	NR	NR	NR	NR	NR	NR	NR	NR	NR
Cerebrovascular disease	NR	NR	NR	NR	NR	NR	NR	NR	NR	NR	SIG	NR	NR	NR
End‐stage renal failure/dialysis	NR	NR	NR	NR	SIG	NR	NR	NS	NR	NR	NR	NR	NR	NR
Chronic kidney disease^c^	NR	NR	NR	NR	NR	NR	NR	NR	NR	NR	SIG	SIG	NR	NR
Retinopathy	NR	NR	NR	NR	NR	NR	NR	NR	NR	NR	SIG	NR	NR	NR
Foot‐related conditions														
Foot infection^d^	NR	NR	NR	NR	NR	NR	SIG	NS	NR	NS	NR	NR	NR	NR
Gangrene	NR	NR	NR	NR	NR	NR	NR	NS	NR	NR	NR	NR	NR	NR
Ulcer site	NR	NR	NR	NR	NR	NR	NS	NR	NR	NR	NR	NR	NR	NR
Ulcer area	NR	NR	NR	NR	NR	NR	NS	NR	NR	NR	NR	NR	NR	NR
Depth of ulcer	NR	NR	NR	NR	NR	NR	NS	NR	NR	NR	NR	NR	NR	NR
Peripheral neuropathy	NR	NR	NR	NR	NR	NR	NS	NR	NR	NR	SIG	SIG	NR	NR
Peripheral artery disease^e^	NR	NR	NR	NR	NR	NR	SIG	SIG	NR	NR	SIG	SIG	NR	NR
Management and other														
Insulin management	NR	NR	NR	NR	SIG	NR	NR	NR	NR	NR	NR	SIG	NR	NR
Oral antidiabetic drugs	NR	NR	NR	NR	SIG	NR	NR	NR	NR	NR	NR	NR	NR	NR
Metformin nonusage	NR	NR	NR	NR	NR	NR	NR	NR	NR	NR	NR	NR	NR	SIG
No professional foot care within 12 months of foot ulcer diagnosis^f^	NR	NR	SIG	NR	NR	NR	NR	NR	NR	NR	NR	NR	SIG	NR
Late referral to specialised foot care	NR	NR	NR	NR	NR	NR	NR	SIG	NR	NR	NR	NR	NR	NR
No multidisciplinary limb preservation program	NR	NR	NR	SIG	NR	NR	NR	NR	NR	NR	NR	NR	NR	NR

Abbreviations: NR, not reported in multivariable analysis; NS, not significant at 95% confidence level; SIG, significant increase.

^a^ Multivariable analysis was not performed in this study.

^b^ Population from the same study. However, Hamilton et al. had many more participants and explored different risk factors for hospitalisation.

^c^ Chronic kidney disease includes eGFR < 60 mL/min and increase In(uACR) mg/mmo.

^d^ Foot infection includes foot infection and MDRB infection (MDRB—multidrug resistance bacteria).

^e^ Peripheral artery disease includes intermittent claudication, ischaemia, history of peripheral revascularisation, pulse pressure (5 mm Hg increase) and absence of any foot pulse.

^f^ No recent professional footcare includes, no preulcerative outpatient foot care within 12 months of foot ulcer diagnosis and no podiatry visits within last 12 months before the ulcer diagnosis (Grouped variables are presented as separate variables with values of effect measure in the detailed Supporting Information S6: Table S5).

In studies of general populations, only diabetes was reported as an independent risk factor [25, 26], whereas in studies of diabetes populations (type 1 and type 2), there were 12 risk factors reported, including male sex, lower education status, type 1 diabetes, cardiovascular disease, end‐stage renal failure, foot infection, PAD, insulin management, oral antidiabetic drug use, no professional footcare within 12 months, late referral to specialised diabetic foot services and no multidisciplinary limb preservation program use [27, 29, 31, 32, 33, 34, 35, 37]. In studies of type 2 diabetes populations specifically, there were 12 reported risk factors, including increased height, increased alcohol consumption, increased HbA1c levels, younger age at diabetes diagnosis, cerebrovascular disease, chronic kidney disease, retinopathy, PAD, peripheral neuropathy, insulin management, metformin nonuse (in those with chronic kidney disease) and no professional footcare within 12 months [24, 28, 30, 36].

Finally, in the seven‐low RoB studies, there were 12 risk factors reported including male sex [34], increased height [28], increased HbA1C [28], diabetes [25, 26], type 1 diabetes [34], younger age at type 2 diabetes diagnosis [28], chronic kidney disease [28], PAD [28], peripheral neuropathy [28], insulin management [28, 33], metformin nonuse (in those with chronic kidney disease) [30] and no professional footcare within 12 months [36].

## 4 Discussion

Our review has synthesised multiple important risk factor findings for foot‐related hospitalisations from 14 eligible studies of nearly 1.8 million community‐dwelling adults. First, nearly all studies have been conducted within diabetes populations, with only two studies in general populations. Second, all studies investigated foot‐related hospitalisation outcomes caused by foot disease, with no other foot‐related conditions investigated. Third, from 97 variables explored across all studies, 22 were reported to be independent risk factors, with eight of those reported in multiple studies. Those eight risk factors were male sex, diabetes, increased HbA1c, chronic kidney disease, peripheral neuropathy, PAD, insulin management and no footcare within 12 months. Fourth, studies assessed as low risk of bias reported those same eight independent risk factors plus increased height, type 1 diabetes and younger age at type 2 diabetes diagnosis. Finally, half of all studies were assessed as being of moderate or high risk of bias and predominantly because of bias in the domains of participation, prognostic measures, attrition, confounding and statistical reporting.

Although this review aimed to identify risk factors for hospitalisation due to any foot‐related condition including musculoskeletal (such as inflammatory arthritis), dermatological (such as ingrown toenails) or traumatic conditions (such as fractures), nearly all eligible studies identified focused on foot‐related hospitalisations caused by foot disease in diabetes populations. Considering previous studies report that around two‐thirds of all foot‐related hospitalisations were caused by foot disease, and half of all foot‐related hospitalisations were in people with diabetes; this focus on foot disease hospitalisations in diabetes populations is perhaps unsurprising [2, 10, 11, 38]. Conversely though, with a half of all foot‐related hospitalisations occurring in people without diabetes and a third of foot‐related hospitalisations caused by other foot‐related conditions [10, 39], this review has identified some important gaps in the existing literature. These gaps may in part be due to difficulty in capturing foot‐related condition characteristics in nondiabetes cohorts or hospitalisations caused by other foot‐related conditions [40]. Regardless, with such a comparatively large hospitalisation burden caused by foot‐related conditions, future studies investigating risk factors for hospitalisations in nondiabetes populations and those caused by other foot‐related conditions are needed.

Our review found eight risk factors for foot disease hospitalisations that were commonly reported in multiple studies and studies of low risk of bias. Those eight risk factors were male sex, diabetes, increased HbA1c, chronic kidney disease, peripheral neuropathy, PAD, insulin management and no footcare within 12 months. Although this is the first review of risk factors for all foot‐related hospitalisations, we note other reviews investigating risk factors for diabetes‐related foot disease development, re‐hospitalisation and amputation outcomes reporting similar risk factors [3, 4, 41, 42, 43, 44, 45, 46, 47]. We suggest that this is unsurprising considering the outcome of hospitalisation fits within the continuum of foot disease development and amputation outcomes. For instance, diabetes, increased HbA1c and insulin management for more complex diabetes, are well‐documented risk factors for the development of foot disease conditions, such as peripheral neuropathy, PAD, ulcers, and infections [17, 41, 48]. These foot disease conditions in turn are also well documented to increase the likelihood of amputation and particularly in those with kidney disease or not receiving footcare [48, 49]. Furthermore, males have been reported to have higher prevalences of diabetes, peripheral neuropathy, PAD and ulcers than females [41, 47]. This increase in foot disease prevalence, along with reported lower self‐care adherence [41, 50] and later attendance to diabetic foot services in males [41, 47], may help explain why males have consistently been found to also have higher amputation rates than females [51]. Thus, whilst the common risk factors we found in this review are perhaps not surprising, considering foot disease is a leading cause of all‐cause hospitalisations, they are still critically important new additions to our knowledge and particularly in those with diabetes.

In addition to the eight commonly reported risk factors, we also identified other risk factors in low risk of bias studies, including type 1 diabetes, younger age at type 2 diabetes diagnosis and increased height to be risk factors. Increased height has been previously implicated in the increased development of neuropathy and hence is found more so in males [50]. Type 1 diabetes and younger age at type 2 diabetes diagnosis have also been previously found to be risk factors for diabetes‐related foot disease development and amputations [46, 52, 53, 54]. This is likely because people with type 1 diabetes are diagnosed at much younger ages and typically have longer disease durations [1, 46]. Somewhat similarly, people diagnosed at younger ages with type 2 diabetes also typically have longer durations of diabetes [1]. However, it is also becoming evident that those with younger onset type 2 diabetes have a more aggressive phenotype of diabetes, particularly for neuropathy [54], and combined with more weight‐bearing activities found in younger people, this has been suggested to produce poorer diabetes‐related foot disease outcomes [55]. Thus, these diabetes factors highlight the importance of introducing targeted preventive strategies in younger populations at risk of foot disease to prevent foot‐related hospitalisations, such as earlier diagnosis, improved glycaemic control and improved guideline‐based footcare access [41, 56].

Although we found half of all eligible studies were at low risk of bias, half were also assessed as having some bias. This was predominantly due to bias in the domains of participation, prognostic measures, attrition, confounding and statistical reporting. Improper identification of source populations and inadequate recruitment of study participants led to most bias we found in the participation domain. Inappropriate measures used for collecting factors and for analysing missing data led to most bias in prognostic measures. Inadequate identification of lost to follow‐up data led to most bias in attrition, whereas improper identification of confounders and inappropriate measures to avoid or minimise confounding effects led to bias in confounding. Finally, inappropriate reporting of the analysis process was identified as the main reason for bias in statistical reporting, including inadequate reporting of regression methods and assumptions checks. Therefore, we recommend such future cohort studies adhere to best practice guidelines for analysing and reporting observational studies to reduce the risk of bias [57, 58, 59].

The findings of this review should be read cognisant of several limitations. First, the search strategy was limited to two databases and studies published after the year 2000, and no handsearching of included study references was performed. Thus, there is a possibility that we may have missed some eligible papers. However, we consider this risk to be small; considering our use of very broad search terms, the two databases have been demonstrated to identify all foot‐related condition literature [13, 14, 15, 16], and two databases are also recommended as appropriate in the AMSTAR best practice systematic review guidelines [60]; a validation set was also used to further validate the search strategy and the high numbers of records screened; plus studies published before 2000 would likely identify different risk factor profiles due to significant changes in footcare practice following the 1999 introduction of international foot‐related clinical guidelines [17]. Second, we did not include grey literature. However, grey literature is challenging to systematically identify in scientific databases, rarely peer reviewed, typically of high risk of bias, and is often not reproducible in peer‐reviewed studies. Third, included studies showed significant heterogeneity in sample size, study settings, factor definitions, outcome definitions and effect measures considered, which precluded us from conducting any meta‐analyses. Last, we included two papers from the same overarching study that had overlapping study populations and exposure variables explored [24, 28]. Although they were analysed as separate studies, this overlap is unlikely to introduce significant bias as we did not perform a meta‐analysis or combine effect estimates across studies.

Conversely, this review has several strengths. First, this review is the first to provide important risk factor evidence on foot‐related hospitalisations. Second, we adhered to our preregistered protocol published in the international prospective register of the systematic reviews database. Third, we tested our search strategy using a validation set; two authors independently screened titles and abstracts, as well as assessed full texts, which enhance the likelihood that we identified all relevant studies. Lastly, a validated risk of bias assessment tool designed and recommended for reviews of risk factors studies was used by two independent authors to ensure that methodological quality was assessed for all included studies.

Findings from this review should help inform practice and policy to potentially prevent foot‐related hospitalisations in future; plus, has identified significant gaps in which further research is required. The common risk factors for foot disease hospitalisation identified in this review were very similar to those previously reported for diabetes‐related foot disease development and amputation outcomes. It is of note that those risk factors have been developed into risk prediction tools for those other foot‐related outcomes [52], and we recommend similar risk prediction tools be explored and developed for foot disease hospitalisation in future as well. Further, with around half of all diabetes‐related foot disease hospitalisations and amputations found to be preventable with guideline‐based care [61], our findings provide direction for preventive strategies to profile and target early identification and treatment of at‐risk populations to potentially reduce high volumes of hospitalisations caused by foot disease in future. Our findings also highlight the need for future robust prospective cohort studies that explore multiple factors for foot‐related hospitalisation in community‐dwelling populations and especially those without diabetes [58, 62]. We recommend that such studies should at least collect the risk factors found in this review to determine if those factors are also risk factors for other foot‐related hospitalisations or if those hospitalisations are precipitated by different risk factor profiles.

### 4.1 Conclusion

This is the first systematic review investigating risk factors for foot‐related hospitalisations among community‐dwelling populations. It has synthesised important findings on common risk factors for hospitalisations caused by foot disease conditions, particularly in diabetes populations; plus, it revealed important gaps in our understanding of hospitalisations caused by other foot‐related conditions and in people without diabetes. Thus, we suggest that people with foot disease and their clinicians should be made aware of these common risk factors, and policymakers should use this knowledge to develop targeted preventive strategies for reducing the risk of hospitalisation for community‐dwelling people with diabetes in particular. We also recommend that future high‐quality cohort studies should investigate risk factors for foot disease‐related hospitalisations in nondiabetes populations, plus risk factors for other foot‐related hospitalisations in general populations.

## Author Contributions

**Sucharitha R. Weerasuriya:** conceptualization, methodology, formal analysis, investigation, writing – original draft, writing – review and editing. **Chanika Alahakoon:** formal analysis, investigation, writing – review and editing. **Nimantha Karunathilaka:** formal analysis, investigation, writing – review and editing. **Yuqi Zhang:** conceptualization, methodology, writing – review and editing, supervision. **Susanna M. Cramb:** conceptualization, methodology, formal analysis, investigation, writing – review and editing, supervision. **Peter A. Lazzarini:** conceptualization, methodology, formal analysis, investigation, writing – review and editing, supervision.

## Funding

S.M.C. and P.A.L. both received funding as National Health and Medical Research Council (NHMRC) Emerging Leadership Investigators (#2008313; #2034266) and S.R.W. received a QUT Postgraduate Research Award Scholarship. This funding organisation had no influence on the conceptualization, design or conduct of the research, nor on the preparation of this paper.

## Ethics Statement

The authors have nothing to report.

## Consent

The authors have nothing to report.

## Conflicts of Interest

The authors declare no conflicts of interest.

## Supporting information


Supporting Information S1

## Data Availability

Data sharing is not applicable to this article as no datasets were generated or analysed during the current study.
