# Supplementary material for: Risk Factors for Foot‐Related Hospitalisations in Adults With and Without Diabetes: A Systematic Review
Source: J Foot Ankle Res. 2026 Jun 8;19(2):e70164. doi: 10.1002/jfa2.70164 (PMC13246321; doi:10.1002/jfa2.70164)
Supplement: Supplementary file 1 — Supporting Information S1 [file JFA2-19-e70164-s001.docx]

**Supplementary material for:**

**Risk factors for foot-related hospitalisations in adults with and without diabetes: a systematic review**

**Table S1:** Validation set of references to validate search strategy

| **No** | **Reference** |
| --- | --- |
| 1 | Henke PK, Blackburn SA, Wainess RW, et al. Osteomyelitis of the foot and toe in adults is a surgical disease: conservative management worsens lower extremity salvage. Ann Surg 2005;241:885–92 |
| 2 | VanGilder C, MacFarlane GD, Harrison P, et al. The demographics of suspected deep tissue injury in the United States: an analysis of the International Pressure Ulcer Prevalence Survey 2006–2009.Adv Skin Wound Care 2010;23:254–61 |
| 3 | Vowden K, Vowden P, Posnett J. The resource costs of wound care in Bradford and Airedale primary care trust in the UK. J Wound Care 2009;18(3):93-4, 6-8, 100 passim |
| 4 | Khammash MR, Obeidat KA, El-Qarqas EA. Screening of hospitalised diabetic patients for lower limb ischaemia: Is it necessary? Singapore Med J 2008;49(2):110-3 |
| 5 | Ozkara A, Delibasi T, Selcoki Y, Fettah Arikan M. The major clinical outcomes of diabetic foot infections: One center experience. Cent Eur J Med 2008;3(4):464-9 |
| 6 | Ogbera AO, Fasanmade O, Ohwovoriole AE, Adediran O. An assessment of the disease burden of foot ulcers in patients with diabetes mellitus attending a Teaching Hospital in Lagos, Nigeria. Int J Low Extrem Wounds 2006;5(4):244-9 |
| 7 | Mohammad Akther J, Ali Khan I, Shahpurkar W, Khanam N, Quazi Syed Z. Evaluation of the diabetic foot according to Wagner's classification in a rural teaching hospital. Br J Diabetes Vase Dis 2011 ;11 (2):74-9 |
| 8 | Lawrence SM, Wraight PR, Campbell DA, Colman PG. Assessment and management of inpatients with acute diabetes-related foot complications: room for improvement. Intern Med J 2004;34(5):229-33 |
| 9 | Chijioke A, Adamu AN, Makusidi AM. Mortality patterns among type 2 diabetes mellitus patients in llorin, Nigeria. Journal of Endocrinology, Metabolism and Diabetes of South Africa 2010;15(2):79—82 |
| 10 | Ajayi EA, Ajayi AO. Pattern and outcome of diabetic admissions at a federal medical center: A 5-year review. Ann Afr Med 2009;8(4):271-5 |
| 11 | Ogbera AO, Chinenye S, Onyekwere A, Fasanmade O. Prognostic indices of diabetes mortality. Ethn Dis 2007;17(4):721-5 |
| 12 | Unachukwu C, Babatunde S, Ihekwaba AE. Diabetes, hand and/or foot ulcers: a cross-sectional hospital-based study in Port Harcourt, Nigeria. Diabetes Res Clin Pract 2007;75(2):148- 52 |
| 13 | Alashek WA, Ehtuish EF. Surgical management and length of hospital stay for diabetic foot. Jamahiriya Med J 2009;9(2):105- 8 |
| 14 | Reed JF, III. An audit of lower extremity complications in octogenarian patients with diabetes mellitus. int J Low Extrem Wounds 2004;3(3):161-4 |
| 15 | Jessup RL. Foot pathology and inappropriate footwear as risk factors for falls in a subacute aged-care hospital. J Am Podiatr Med Assoc 2007;97(3):213-7 |
| 16 | Kaminski M, Frescos N, Tucker S. Prevalence of risk factors for foot ulceration in patients with end-stage renal disease on haemodialysis. Intern Med J 2012;42(6):e120-8 |
| 17 | Monge L, Gnavi R, Carnà P, Broglio F, Boffano GM, Giorda CB. Incidence of hospitalization and mortality in patients with diabetic foot regardless of amputation: a population study. Acta Diabetologica. 2020;57:221-8 |
| 18 | Lavery LA, Armstrong DG, Wunderlich RP, Mohler MJ, Wendel CS, Lipsky BA. Risk factors for foot infections in individuals with diabetes. Diabetes Care. 2006 Jun;29(6):1288-93. doi: 10.2337/dc05-2425. PMID: 16732010 |
| 19 | Baba M, Davis WA, Davis TM. A longitudinal study of foot ulceration and its risk factors in community-based patients with type 2 diabetes: the Fremantle Diabetes Study. Diabetes Res Clin Pract. 2014;106:42-9 |
| 20 | Fang M, Ishigami J, Echouffo-Tcheugui JB, Lutsey PL, Pankow JS, Selvin E. Diabetes and the risk of hospitalisation for infection: the Atherosclerosis Risk in Communities (ARIC) study. Diabetologia. 2021;64:2458-65 |
| 21 | Quigley M, Morton JI, Lazzarini PA, Zoungas S, Shaw JE, Magliano DJ. Trends in diabetes-related foot disease hospitalizations and amputations in Australia, 2010 to 2019. Diabetes Research and Clinical Practice. 2022;194 |

**Table S2:** Search strategy used for electronic databases

| **PubMed** |
| --- |
| "foot"[MeSH] OR (foot OR feet)[TIAB]  AND  (‘foot injuries’ OR ‘diabetic foot ‘OR ‘foot ulcer’ OR ‘foot disease’ OR ‘foot dermatoses’ OR ‘foot deformities’ OR surgical OR fracture*)[MeSH] OR (isch* OR amput* OR surgical OR diab* OR arthritis*)[All field] OR (‘foot injr* OR ‘diabetic foot’ OR ‘foot ulcer’ OR ‘foot diseas*’ OR ‘foot dermatos*’ OR ‘foot deformit*’ OR ‘foot problem*’ OR ‘foot complication*’ OR wound* Or ‘foot infec*’ OR cellulitis OR gangr* OR arter* OR vascul* OR isch* OR neurop* OR amputation* OR diab* OR arthrit* OR musc* OR fracture* OR ‘foot deformit*’ OR tendon* OR trauma* OR dermatology*) [TIAB]  AND  (Predictors OR factor OR risk OR causality OR Indicators) [MeSH] OR (prdict* OR factor* OR risk* OR predispose* OR contribut* OR associate* OR relat* OR correlate* OR etiolog* OR aetiolog* OR develop* OR demograph* OR indicator* OR caus*)[TIAB]  AND  (hospital OR inpatient) [MeSH] OR (hospital* OR inpatient* OR admin* OR admit* ) [TIAB] |
| **Embase** |
| (foot OR feet): ti,ab  AND  (‘foot disease’ OR ‘foot infection’ OR ‘foot injury’ OR ‘foot ulcer’ OR ‘diabetic foot’ OR ‘foot malformation’ OR amputation OR ‘diabetes mellitus’ OR arthritis OR fracture OR ‘wounds and injuries’ OR ):exp OR (foot diseas*’ OR ‘foot infec*’ OR ‘foot injur*’ OR ‘foot ulcer*’ OR ‘diabetic foot’ OR ‘foot malformation*’ OR amputation* OR ‘diabetes mellitus’ OR arthritis OR fracture* OR ‘foot problem*’ OR ‘foot complication*’ OR wound* OR cellulitis OR isch* OR vascul* OR arter* OR gangr* OR neurop* OR amputation* OR diab* OR arthrit* OR musc* OR “foot deform*’ OR tendon* OR trauma* OR dermatology*):ti,ab  AND  Causality: exp OR (predict* OR factor* OR risk* OR predispose* OR contribut* OR associate* OR relat* OR correlate* OR etiolog* OR aetiolog* OR caus* OR develop* OR demograph* OR indicator*): ti,ab  AND  (hospital* OR inpatient* OR admin* OR admit*): ti,ab |

**Table S3:** Agreed criteria for rating risk of bias domains using the QUIPS tool for this systematic review

| **1. Study Participation** | **Agreed criteria** |
| --- | --- |
| Source of target population | Any information available on gender, age and disease-related information. |
| Method used to identify population | Information available on the patients’ recruitment (from which health service) together with a description of how the data collection was performed. |
| Recruitment period | There should be information available on the beginning and end of the data collection, the setting, and the name of a geographical place or hospital, follow up period. |
| Place of recruitment |  |
| Inclusion and exclusion criteria | At least 1 inclusion and 1 exclusion criterion should be given. |
| Adequate study participation | Not all the eligible people will participate in a follow up study. Therefore, adequate participation is defined as >67%. ^(1)^   1. Grooten WJA, Tseli E, Äng BO, Boersma K, Stålnacke B-M, Gerdle B, Enthoven P. Elaborating on the assessment of the risk of bias in prognostic studies in pain rehabilitation using QUIPS—aspects of interrater agreement. Diagnostic and Prognostic Research. 2019;3(1):5. |
| Baseline characteristics | Basic information available regarding gender, age, socioeconomic status together with some conditions related information (ulcer, infection, comorbidities) and information on relevant outcome data. |
| **Summary Study participation** |  |
|  |  |
| **2. Study Attrition** |  |
| Proportion of baseline sample available for analysis | Adequate participation defined as > 67%. ^(1)^   1. Grooten WJA, Tseli E, Äng BO, Boersma K, Stålnacke B-M, Gerdle B, Enthoven P. Elaborating on the assessment of the risk of bias in prognostic studies in pain rehabilitation using QUIPS—aspects of interrater agreement. Diagnostic and Prognostic Research. 2019;3(1):5. |
| Attempts to collect information on participants who dropped out | Information available on the methods and timing. |
| Reasons and potential impact of subjects lost to follow-up | Any information available on the reasons for dropouts. |
| Outcome and prognostic factor information on those lost to follow-up  No difference drop-outs and completers | Any information available on gender, age and disease-related information for dropouts. |
|  | There should be no differences between the participants and non-participants in regard to demographic and illness-related variables. |
| **Study Attrition Summary** |  |
|  |  |
| **3. Prognostic Factor Measurement** |  |
| Definition of the PF | There should be a clear definition of the PF, e.g. information on which question(s) were used, how the data was collected, how the variable was constructed, etc |
| Valid and Reliable Measurement of PF  Continuous variables or cut-points | Clearly mentioned whether the PF measured with a valid and reliable instrument or validation study of the instrument was done before doing the proper study. |
|  | The cut-offs used should NOT be based on distribution of the data, but on established cut-offs in the field of foot related conditions. |
| Method and Setting of PF Measurement | If more than one instrument use to measure any PF, all instruments used should be valid and reliable. |
| Proportion of data on PF available for analysis | There should be at least 67% ^(1)^ available with complete data. It is important also to check if there is different data available for different prognostic factors measured simultaneously, which could indicate differential loss-to follow-up.   1. Grooten WJA, Tseli E, Äng BO, Boersma K, Stålnacke B-M, Gerdle B, Enthoven P. Elaborating on the assessment of the risk of bias in prognostic studies in pain rehabilitation using QUIPS—aspects of interrater agreement. Diagnostic and Prognostic Research. 2019;3(1):5. |
| Method used for missing data | There should be some kind of imputation, but even if no imputation was done, it could be a “yes” if at least 67% of the study sample had complete data. |
| **PF Measurement Summary** |  |
|  |  |
| **4. Outcome Measurement** |  |
| Definition of the Outcome | There should be a clear definition of the outcome measure available, e.g. information on the question(s) used, how the data was collected, how the variable was constructed, etc. |
| Valid and Reliable Measurement of Outcome | There should be a reference to a reliability/validity study or information on these features in the paper. Note the population on which the reliability/validity study was performed should correspond to the population of interest, in this case patients with foot related condition and citation of a reliable source. |
| Method and Setting of Outcome Measurement | The outcome measures should be the same for all the study participants as our outcome is hospitalisation. If there are multicentre studies, outcome measured in different centres should be same. |
| **Outcome Measurement Summary** |  |
|  |  |
| **5. Study Confounding** |  |
| Important Confounders Measured | There should be at least one confounder taken into account other than age/sex to be rated as 'yes'. If only Age or sex or both considered as confounders this sub domain would be rated as 'partial'. |
| Definition of the confounding factor | There should be a clear definition of the confounder measure available, e.g. information on the question(s) used, how the data was collected, how the variable was constructed, etc. |
| Valid and Reliable Measurement of Confounders | There could be a reference to a reliability/validity study or information on these features in the paper and the confounder should be valid for the use in the field of foot related conditions. |
| Method and Setting of Confounding Measurement | The confounder measures should be the same for all participants. |
| Method used for missing data | There could be any kind of imputation, but even if no imputation was done, it could be a “yes” if at least 67% ^(1)^ of the study sample had complete data.   1. Grooten WJA, Tseli E, Äng BO, Boersma K, Stålnacke B-M, Gerdle B, Enthoven P. Elaborating on the assessment of the risk of bias in prognostic studies in pain rehabilitation using QUIPS—aspects of interrater agreement. Diagnostic and Prognostic Research. 2019;3(1):5. |
| Appropriate Accounting for Confounding  Appropriate Accounting for in analysis | There should have been some kind of randomization or stratification for confounders included in the analyses. |
|  | There should be some form of statistical analyses description available, resulting in information on the effect of the PF on the outcome. |
| **Study Confounding Summary** |  |
|  |  |
| **6. Statistical Analysis and Reporting** |  |
| Presentation of analytical strategy | There should be enough information available to understand the statistical methods applied, so that the rater can determine whether the methods used were correct. (points 1-15 in the SAMPL guideline) if 15/15 classified as ‘yes’ 0/15 classified as ‘no’ or ‘unsure’ otherwise classified as ‘partial’ if articles report rates total count should be 20 (points 1-20 in the SAMPL guideline). 20/20 ‘yes’, 0/20 ‘no’ or ‘unsure’ otherwise ‘partial’. |
| Model development strategy  Statistical model adequate for study design | There should be some form of statistical analyses description available, resulting in information on the effect of the PF on the outcome (points 21-35/ 36-45/ 46-57 according to the relevant analysis in the SAMPL guideline). If all points present classified as ‘yes’ if none of the points present classified as ‘no’ or ‘unsure’ otherwise classified as ‘partial’. ^(2)^   1. Lang TA, Altman DG. Basic statistical reporting for articles published in biomedical journals: the "Statistical Analyses and Methods in the Published Literature" or the SAMPL Guidelines. Int J Nurs Stud. 2015;52(1):5-9. |
|  |  |
| Reporting of results | All variables (outcomes and PF) that are described in the method section should be included in the result section with words or in numbers (tables, figures). |
| **Statistical Analysis and Presentation Summary** |  |

QUIPS- Quality in Prognostic Studies

**Table S4:** Criteria of SAMPL guideline considered for statistical analysis and reporting domain of the QUIP tool

|  | **Primary analyses** |
| --- | --- |
|  |  |
| 01 | Describe the purpose of the analysis |
| 02 | Identify the variables used in the analysis and summarize each with descriptive statistics |
| 03 | Describe fully the main methods for analyzing the primary objectives of the study |
| 04 | Make clear which method was used for each analysis |
| 05 | Verify that that data conformed to the assumptions of the test used to analyze them |
| 06 | If relevant, report how any outlying data were treated in the analysis |
| 07 | Say whether tests were one- or two-tailed and justify the use of one-tailed tests |
| 08 | Report the alpha level (e.g., 0.05) that defines statistical significance |
| 09 | Name the statistical package or program used in the analysis |
|  |  |
|  | **Reporting numbers and descriptive statistics** |
|  |  |
| 10 | Report numbers—especially measurements—with an appropriate degree of precision. For ease of comprehension and simplicity, round to a reasonable extent. |
| 11 | Report total sample and group sizes for each analysis. |
| 12 | Report numerators and denominators for all percentages |
| 13 | Summarize data that are approximately normally distributed with means and standard deviations (SD). |
| 14 | Do NOT use the standard error of the mean (SE) to indicate the variability of a data set |
| 15 | Display data in tables or figures. |
|  |  |
|  | **Reporting risk, rates, and ratios** |
|  |  |
| 16 | Identify the type |
| 17 | Identify the quantities represented in the numerator and denominator (rates) |
| 18 | Identify the time period over with each rate applies |
| 19 | Identify any unit of population (that is, the unit multiplier: e.g., x 100; x 10,000) associated with the rate. |
| 20 | Consider reporting a measure of precision (a confidence interval) for estimated risks, rates, and ratios. |
|  |  |
|  | **Reporting regression analyses** |
|  |  |
| 21 | Describe the purpose of the analysis |
| 22 | Identify the variables used in the analysis and summarize each with descriptive statistics |
|  |  |
| 23 | Confirm that the assumptions of the analysis were met |
| 24 | If relevant, report how any outlying values were treated in the analysis. |
| 25 | Report how any missing data were treated in the analyses |
| 26 | For either simple or multiple (multivariable) regression analyses, report the regression equation. |
| 27 | For multiple regression analyses: 1) report the alpha level used in the univariate analysis; |
| 28 | For multiple regression analyses: 2) report whether the variables were assessed for a) collinearity |
| 29 | For multiple regression analyses: 2) report whether the variables were assessed for b) interaction |
| 30 | For multiple regression analyses: 3) describe the variable selection process by which the final model was developed (e.g., forward stepwise; best subset) |
| 31 | Report the regression coefficients (beta weights) of each explanatory variable and the associated confidence intervals and P values, preferably in a table. |
| 32 | Provide a measure of the model's "goodness-of-fit" to the data |
| 33 | Specify whether and how the model was validated |
| 34 | For primary comparisons analysed with simple linear regression analysis, consider reporting the results graphically, in a scatter plot showing the regression line and its confidence bounds. Do not extend the regression line (or the interpretation of the analysis) beyond the minimum and maximum values of the data. |
| 35 | Name the statistical package or program used in the analysis |
|  |  |
|  | **Reporting analyses of variance (ANOVA) or of covariance (ANCOVA)** |
|  |  |
| 36 | Describe the purpose of the analysis |
| 37 | Identify the variables used in the analysis and summarize each with descriptive statistics |
| 38 | Confirm that the assumptions of the analysis were met. For example, indicate whether an analysis of residuals confirmed the assumptions of linearity |
| 39 | If relevant, report how any outlying data were treated in the analysis |
| 40 | Report how any missing data were treated in the analyses |
| 41 | Specify whether the explanatory variables were tested for interaction, and if so how these interactions were treated |
| 42 | If appropriate, in a table, report the *P* value for each explanatory variable, the test statistics and, where applicable, the degrees of freedom for the analysis |
| 43 | Provide an assessment of the goodness-of-fit of the model to the data, such as *R*2. |
| 44 | Specify whether and how the model was validated |
| 45 | Name the statistical package or program used in the analysis |
|  |  |
|  | **Reporting survival (time-to-event) analyses** |
|  |  |
| 46 | Describe the purpose of the analysis |
| 47 | Identify the dates or events that mark the beginning, and the end of the time period analysed. |
| 48 | Specify the circumstances under which data were censored |
|  |  |
| 49 | Specify the statistical methods used to estimate the survival rate |
| 50 | Confirm that the assumptions of survival analysis were met |
| 51 | For each group, give the estimated survival probability at appropriate follow-up times, with confidence intervals, and the number of participants at risk for death at each time. |
| 52 | Reporting median survival times, with confidence intervals, is often useful to allow the results to be compared with those of other studies |
| 53 | Consider presenting the full results in a graph |
| 54 | Specify the statistical methods used to compare two or more survival curves |
| 55 | When comparing two or more survival curves with hypothesis tests, report the P value of the comparison |
| 56 | Report the regression model used to assess the associations between the explanatory variables and survival or time-to-event |
| 57 | Report a measure of risk (e.g., a hazard ratio) for each explanatory variable, with a confidence interval |
|  |  |

SAMPL Statistical Analyses and Methods in the Published Literature, QUIPS- Quality in Prognostic Studies

**Table S5:** Exposure variables reported in included studies

|  | Dinh et al., 2022 | Fang et al., 2021 | Gibson et al., 2013 | Manji et. al., 2024 | Monge et.al., 2019 | Quigley et. al., 2022 | Ha Van et al., 2023 | Meloni et.al., 2021 | Schimidt et. al., 2023 | Yovera et al., 2024 | Baba et al., 2014** | Hamilton et al., 2021** | Tan et al., 2024 | Kwon et al., 2024 |
| --- | --- | --- | --- | --- | --- | --- | --- | --- | --- | --- | --- | --- | --- | --- |
| **Study setting** | PB | PB | PB | PB | PB | PB | MC | SC | SC | SC | PB | PB | PB | MC |
| **Population of interest** | General | General | Diabetes | | | | | | | | Type 2 diabetes | | | Type 2 diabetes with kidney disease |
| **Sociodemographic variables** |  |  |  |  |  |  |  |  |  |  |  |  |  |  |
| Age | ✓ | ✓ | ✓ |  |  | ✓ | ✓ | ✓ | ✓ | ✓ | ✓ | ✓ | ✓ | ✓ |
| Sex /gender | ✓ | ✓ | ✓ |  |  | ✓ | ✓ | ✓ | ✓ | ✓ | ✓ | ✓ | ✓ | ✓ |
| Race |  | ✓ |  |  |  |  |  |  | ✓ |  |  |  |  |  |
| Ethical background |  |  |  |  |  |  |  |  |  |  | ✓ | ✓ |  |  |
| Marital status |  |  |  |  |  |  |  |  |  |  | ✓ | ✓ |  |  |
| Geographical Residence/ Urban |  |  | ✓ |  |  |  |  |  |  |  |  |  |  |  |
| Health insurance status/type |  | ✓ | ✓ |  |  |  |  |  |  |  |  |  |  |  |
| Household income |  | ✓ | ✓ |  |  |  |  |  |  |  |  |  |  |  |
| Education level |  | ✓ | ✓ |  | ✓ |  |  |  |  |  | ✓ | ✓ |  |  |
| Height (1cm increase) |  | ✓ |  |  |  |  |  |  |  |  | ✓ | ✓ |  |  |
| Smoking status |  | ✓ |  |  |  |  |  |  | ✓ |  | ✓ | ✓ |  | ✓ |
| Alcohol consumption |  | ✓ |  |  |  |  |  |  |  |  | ✓ | ✓ |  |  |
| Employment status |  | ✓ | ✓ |  |  |  |  |  |  |  |  |  |  |  |
| Socio-economic status |  | ✓ |  |  |  |  |  |  |  |  |  |  |  |  |
| Exercise in past weeks |  |  |  |  |  |  |  |  |  |  | ✓ |  |  |  |
|  |  |  |  |  |  |  |  |  |  |  |  |  |  |  |
| **Biomedical measures** |  |  |  |  |  |  |  |  |  |  |  |  |  |  |
| BMI/ Obesity |  | ✓ |  |  |  |  | ✓ |  | ✓ |  | ✓ | ✓ | ✓ | ✓ |
| Total cholesterol |  | ✓ |  |  |  |  |  |  |  |  | ✓ | ✓ |  |  |
| HDL cholesterol |  | ✓ |  |  |  |  |  |  |  |  | ✓ | ✓ |  |  |
| LDL cholesterol |  | ✓ |  |  |  |  |  |  |  |  | ✓ |  |  |  |
| Triacylglycerol |  | ✓ |  |  |  |  |  |  |  |  | ✓ | ✓ |  |  |
| Fasting blood glucose level |  |  |  |  |  |  |  |  |  |  | ✓ | ✓ |  |  |
| HbA1c |  |  |  |  |  |  | ✓ | ✓ | ✓ | ✓ | ✓ | ✓ |  | ✓ |
| Past history of self-reported hypoglycemia |  |  |  |  |  |  |  |  |  |  | ✓ | ✓ |  |  |
| WBC |  |  |  |  |  |  |  |  | ✓ |  |  |  |  |  |
| Systolic blood pressure (mmHg) |  |  |  |  |  |  |  |  |  |  | ✓ | ✓ |  |  |
| Diastolic blood pressure (mmHg) |  |  |  |  |  |  |  |  |  |  | ✓ | ✓ |  |  |
| Hypertension |  | ✓ |  |  |  |  |  | ✓ |  |  |  |  | ✓ | ✓ |
| Creatinine |  |  |  |  |  |  |  |  |  |  |  |  |  | ✓ |
| Albumin |  |  |  |  |  |  |  |  |  | ✓ |  |  |  | ✓ |
| Heamoglobin |  |  |  |  |  |  |  |  |  | ✓ |  |  |  |  |
| eGFR |  | ✓ |  |  |  |  |  |  | ✓ |  | ✓ | ✓ |  | ✓ |
| uACR (mg/mmol) |  |  |  |  |  |  |  |  |  |  | ✓ | ✓ |  |  |
|  |  |  |  |  |  |  |  |  |  |  |  |  |  |  |
| **Comorbidities** |  |  |  |  |  |  |  |  |  |  |  |  |  |  |
| Diabetes | ✓ |  |  |  |  |  |  |  |  |  |  |  | ✓ |  |
| Diabetes type |  |  |  |  | ✓ | ✓ |  | ✓ | ✓ |  |  |  |  |  |
| Age at diabetes diagnosis |  |  |  |  |  | ✓ |  |  |  |  |  | ✓ |  |  |
| Duration of diabetes diagnosis |  |  |  |  |  |  | ✓ | ✓ |  | ✓ | ✓ | ✓ |  |  |
| Cardiovascular disease/ Chronic Heart Disease |  | ✓ | ✓ |  | ✓ |  |  | ✓ | ✓ |  | ✓ |  | ✓ | ✓ |
| Atrial fibrillation |  |  |  |  |  |  |  |  |  |  |  | ✓ |  |  |
| Hospitalisation for/with heart failure |  |  |  |  |  |  |  |  |  |  |  | ✓ | ✓ |  |
| Myocardial infarction |  |  |  |  |  |  |  |  |  |  |  | ✓ |  |  |
| Ischaemic heart disease |  |  |  |  |  |  |  | ✓ |  |  | ✓ | ✓ |  |  |
| Cerebrovascular disease |  |  |  |  | ✓ |  |  | ✓ |  |  | ✓ | ✓ | ✓ | ✓ |
| Stroke |  |  |  |  |  |  |  |  |  |  | ✓ | ✓ |  |  |
| Hypercholesterolemia/ Dyslipidaemia |  |  |  |  |  |  |  | ✓ |  |  |  |  |  | ✓ |
| End stage renal failure/dialysis |  |  |  |  | ✓ |  | ✓ | ✓ |  |  |  |  | ✓ |  |
| Chronic kidney disease |  |  |  |  | ✓ |  |  |  | ✓ | ✓ |  |  | ✓ |  |
| Nephropathy |  |  | ✓ |  |  |  | ✓ |  |  |  |  |  |  |  |
| Retinopathy |  |  | ✓ |  |  |  |  |  |  | ✓ | ✓ |  |  |  |
| COPD |  |  |  |  |  |  |  | ✓ |  |  |  |  | ✓ |  |
| Liver disease |  |  |  |  |  |  |  |  |  |  |  |  | ✓ | ✓ |
| Depression |  |  |  |  |  |  |  |  |  |  |  |  | ✓ |  |
|  |  |  |  |  |  |  |  |  |  |  |  |  |  |  |
| **Foot-related conditions** |  |  |  |  |  |  |  |  |  |  |  |  |  |  |
| Ulceration/ History of ulcer |  |  |  |  |  | ✓ | ✓ |  |  | ✓ | ✓ |  |  | ✓ |
| Uncomplicated DFU |  |  |  |  |  |  |  | ✓ |  |  |  |  |  |  |
| Complicated DFU |  |  |  |  |  |  |  | ✓ |  |  |  |  |  |  |
| Severely complicated DFU |  |  |  |  |  |  |  | ✓ |  |  |  |  |  |  |
| Foot infection |  |  |  |  |  |  | ✓ | ✓ |  | ✓ |  |  |  |  |
| MDRB infection |  |  |  |  |  |  |  |  |  | ✓ |  |  |  |  |
| Cellulitis |  |  |  |  |  | ✓ |  |  |  |  |  |  |  |  |
| Gangrene |  |  |  |  |  |  |  | ✓ |  |  |  |  |  |  |
| Ulcer site |  |  |  |  |  |  | ✓ |  |  | ✓ |  |  |  |  |
| Ulcer area/dimension |  |  |  |  |  |  | ✓ | ✓ |  | ✓ |  |  |  |  |
| Depth of ulcer |  |  |  |  |  |  | ✓ | ✓ |  | ✓ |  |  |  |  |
| Ischaemia |  |  |  |  |  |  | ✓ |  |  | ✓ |  |  |  |  |
| Topography ((surface of foot with ulcers) |  |  |  |  |  |  |  |  |  | ✓ |  |  |  |  |
| Oedema |  |  |  |  |  |  |  |  |  | ✓ |  |  |  |  |
| Degree of injury inflammation |  |  |  |  |  |  |  |  |  | ✓ |  |  |  |  |
| Number of affected zones/number of ulcers |  |  |  |  |  |  |  |  |  | ✓ |  |  |  |  |
| Osteomyelitis |  |  |  |  |  | ✓ |  |  | ✓ |  |  |  |  |  |
| Peripheral Neuropathy |  |  | ✓ |  |  | ✓ |  |  |  | ✓ | ✓ | ✓ |  |  |
| Peripheral Artery Disease (PAD) |  |  | ✓ |  |  | ✓ |  | ✓ |  | ✓ | ✓ | ✓ | ✓ |  |
| Intermittent claudication |  |  |  |  |  |  |  |  |  |  | ✓ | ✓ |  |  |
| Pulse pressure |  |  |  |  |  |  |  |  |  |  | ✓ |  |  |  |
| Pulse rate |  |  |  |  |  |  |  |  |  |  |  | ✓ |  |  |
| Absence of pulse pressure |  |  |  |  |  |  |  |  |  |  |  | ✓ |  |  |
| Peripheral revascularization |  |  |  |  |  |  |  |  |  |  | ✓ | ✓ |  |  |
| Toe brachial index |  |  |  |  |  |  |  |  | ✓ |  |  |  |  |  |
| Toe pressure |  |  |  |  |  |  |  |  | ✓ |  |  |  |  |  |
| Pedal pulses |  |  |  |  |  |  |  |  |  |  | ✓ |  |  |  |
| Ankle brachial index |  |  |  |  |  |  |  |  |  |  | ✓ |  |  |  |
| Callus and others (e.g. abrasions) |  |  | ✓ |  |  |  |  |  |  |  |  |  |  |  |
| History of amputation |  |  |  |  |  |  |  |  | ✓ | ✓ |  |  |  |  |
|  |  |  |  |  |  |  |  |  |  |  |  |  |  |  |
| **Management and service related** |  |  |  |  |  |  |  |  |  |  |  |  |  |  |
| Diet |  |  |  |  | ✓ |  |  |  |  |  | ✓ | ✓ |  |  |
| Insulin therapy |  |  |  |  | ✓ |  | ✓ |  |  |  | ✓ | ✓ |  |  |
| Oral antidiabetes drugs |  |  |  |  | ✓ |  |  |  |  |  | ✓ | ✓ |  | ✓ |
| Metformin non-usage |  |  |  |  |  |  |  |  |  |  |  |  |  | ✓ |
| Antihypertensive medication |  |  |  |  |  |  |  |  |  |  | ✓ | ✓ |  |  |
| Lipid-modifying medication |  |  |  |  |  |  |  |  |  |  | ✓ | ✓ |  |  |
| Aspirin use |  |  |  |  |  |  |  |  |  |  | ✓ | ✓ |  |  |
| Previous antibiotic therapy |  |  |  |  |  |  |  |  |  | ✓ |  |  |  |  |
| Professional foot care within 12 months of foot ulcer diagnosis |  |  |  |  |  |  |  |  |  |  |  |  | ✓ |  |
| Past Podiatry appointments |  |  |  |  |  |  |  |  |  |  | ✓ |  |  |  |
| Prior diabetes education |  |  |  |  |  |  |  |  |  |  | ✓ |  |  |  |
| Late referral to specialised foot care |  |  |  |  |  |  |  | ✓ |  |  |  |  |  |  |
| Multidisciplinary limb preservation program |  |  |  | ✓ |  |  |  |  |  |  |  |  |  |  |
| Charlson comorbidity index | ✓ |  | ✓ |  |  |  |  |  | ✓ |  |  |  | ✓ |  |
| Psychiatric diagnosis groups |  |  | ✓ |  |  |  |  |  |  |  |  |  |  |  |
| Medication possession ratio |  |  | ✓ |  |  |  |  |  |  |  |  |  |  |  |

✓ Green- Reported in the text or a table, Grey- Not reported in univariate/bivariate analysis, PB-Population based, MC- Multi-centre, SC- Single centre, BMI- Body mass index, HDL- High density lipoproteins, LDL- Low density lipoproteins , HbA1c – glycated haemoglobin, WBC- white blood cell, eGFR- Estimated glomerular filtration rate, uACR- Urine albumin- creatinine ratio, COPD- Chronic obstructive pulmonary disease, DFU- Diabetes foot ulcer, MDRB- Multi Drug Resistance bacteria, , **Population from same study, however Hamilton et.al. had many more participants and explore different risk factors for hospitalisation

**Table S6:** Detailed risk factor findings for foot-related hospitalisation reported in included studies

| **Reference** | | **Dinh et al., 2022** | **Fang et al., 2021** | **Gibson et al., 2013** | **Manji et.al., 2024** | **Monge et.al., 2019** | **Quigley et. al., 2022** | **Ha Van et al., 2023** | **Meloni et.al., 2021** | **Schimidt et. al., 2023^$^** | **Yovera et al., 2024** | **Baba et al., 2014****** | **Hamilton et al., 2021****** | **Tan et al., 2024** | **Kwon et al., 2024** |  |
| --- | --- | --- | --- | --- | --- | --- | --- | --- | --- | --- | --- | --- | --- | --- | --- | --- |
|  | |  |  |  |  |  |  |  |  |  |  |  |  |  |  |  |
| Population participated | | General population with kidney disease | General population (Diabetes and no diabetes patients) | Diabetes patients | Diabetes patients | Diabetes patients | Diabetes patients | Diabetes patients | Diabetes patients | Diabetes patients | Diabetes patients | Type 2 diabetes patients only | Type 2 diabetes patients only | Type 2 diabetes patients only | Type 2 diabetes patients with kidney disease |  |
|  | |  |  |  |  |  |  |  |  |  |  |  |  |  |  |  |
| Effect measure | | Age adjusted incidence rate per 10,000 person years | Adjusted hazard ratio | Weighted adjusted hazard ratio | Incident rate per 100 diabetes population | Age adjusted incidence rate per 100,000 population | Age adjusted incidence rate per 1,000 person years | Odds ratio | Odds ratio | Odds ratio | Odds ratio | Adjusted hazard ratio | Cause specific hazard ratio | Odds ratio | Adjusted hazard ratio |  |
| Study setting | | PB | PB | PB | PB | PB | PB | MC | SC | SC | SC | PB | PB | PB | MC |  |
| **Sociodemographic variables** | | |  |  |  |  |  |  |  |  |  |  |  |  |  |  |
| Sex | | NR | NR | NR | NR | SIG M: 2178.8 (2095.6–2261.9) F: 1352.3 (1287.5-1417.1) | SIG T1 M: 36.74 (34.71, 38.77) F: 22.77 (20.97, 24.56) T2 M: 31.24 (30.72, 31.75) F: 17.04 (16.61, 17.46) | NR | NR | NR | NR | NR | NR | NR | NR |  |
| Education level | | NR | NR | NR | NR | SIG | NR | NR | NR | NR | NR | NR | NR | NR | NR |  |
| High | |  |  |  |  | M: 2098.9 (1896.4–2301.4) F: 1089.3 (883.2–1295.4) |  |  |  |  |  |  |  |  |  |  |
| Medium | |  |  |  |  | M: 2308.8 (2152.2- 2465.3) F: 1482.3(1324.8- 1639.8) |  |  |  |  |  |  |  |  |  |  |
| Low | |  |  |  |  | M: 2595 (2448- 2743) F: 1551 (1458-1645) |  |  |  |  |  |  |  |  |  |  |
| Height (1cm increase) | | NR | NR | NR | NR | NR | NR | NR | NR | NR | NR | NR | SIG 1.04 (1.01–1.08) | NR | NR |  |
| Alcohol consumption (1stand/day) | | NR | NR | NR | NR | NR | NR | NR | NR | NR | NR | SIG 1.16 (1.05–1.27) | NR | NR | NR |  |
| **Biomedical measures** | | |  |  |  |  |  |  |  |  |  |  |  |  |  |  |
| HbA1c (1% increase) | NR | | NR | NR | NR | NR | NR | NR | NR | NR | NR | SIG 1.2 (1.1–1.4) | SIG 1.2 (1.0–1.4) | NR | NR |  |
| **Comorbidities** | |  |  |  |  |  |  |  |  |  |  |  |  |  |  |  |
| Diabetes Vs no diabetes | | SIG | SIG | NR | NR | NR | N R | NR | NR | NR | NR | NR | NR | NR | NR |  |
|  | |  | 9.0 (6.7-12.0)+ |  |  |  |  |  |  |  |  |  |  |  |  |  |
|  | |  | 8.3(6.2-12.0)++ |  |  |  |  |  |  |  |  |  |  |  |  |  |
|  | |  | 6.0 (4.4-8.2)+++ |  |  |  |  |  |  |  |  |  |  |  |  |  |
| Diabetes | | 173.3 (154.4, 194.4) |  |  |  |  |  |  |  |  |  |  |  |  |  |  |
| No diabetes | | 9.0 (7.2, 11.2) |  |  |  |  |  |  |  |  |  |  |  |  |  |  |
| Type 1 Vs Type 2 diabetes | | NR | NR | NR | NR | NR | SIG | NR | NR | NR | NR | NR | NR | NR | NR |  |
| Male | |  |  |  |  |  | T1: 36.74 (34.71, 38.77) T2: 31.24 (30.72, 31.75) |  |  |  |  |  |  |  |  |  |
| Female | |  |  |  |  |  | T1: 22.77 (20.97, 24.56) T2: 17.04 (16.61, 17.46) |  |  |  |  |  |  |  |  |  |
| Age at diabetes diagnosis increase in one year | | NR | NR | NR | NR | NR | NR | NR | NR | NR | NR | NR | SIG 0.96 (0.94–0.99) | NR | NR |  |
| Cardiovascular disease | | NR | NR | NR | NR | SIG | NR | NR | NR | NR | NR | NR | NR | NR | NR |  |
| Yes | |  |  |  |  | M: 3930.5(3612.2–4248.9) F: 2790.0(2451.5–3128.4) |  |  |  |  |  |  |  |  |  |  |
| No | |  |  |  |  | M: 1957.7 (1873.4–2042.0) F: 1217.8(1154.4–1281.3) |  |  |  |  |  |  |  |  |  |  |
| Cerebrovascular disease | | NR | NR | NR | NR | NR | NR | NR | NR | NR | NR | SIG 3.76 (1.97–7.19) | NR | NR | NR |  |
| End stage renal failure | | NR | NR | NR | NR | NR | NR | NR | NS 0.8 (0.5–1.7) | NR | NR | NR | NR | NR | NR |  |
| Previous dialysis | | NR | NR | NR | NR | SIG | NR | NR | NR | NR | NR | NR | NR | NR | NR |  |
| Yes | |  |  |  |  | M: 14,114.4 (11,521–16,708) F: 12,779.5 (9668.1–15,891) |  |  |  |  |  |  |  |  |  |  |
| No | |  |  |  |  | M: 2124.5 (2042.2–2206.9) F: 1290.5 (1227.6–1353.3) |  |  |  |  |  |  |  |  |  |  |
| **Chronic kidney disease** | |  |  |  |  |  |  |  |  |  |  |  |  |  |  |  |
| eGFR<60mL/min | | NR | NR | NR | NR | NR | NR | NR | NR | NR | NR | SIG 2.12 (1.30–3.51) | NR | NR | NR |  |
| In(uACR) mg/mmo | | NR | NR | NR | NR | NR | NR | NR | NR | NR | NR | NR | SIG 1.40 (1.17–1.67) | NR | NR |  |
| Retinopathy | | NR | NR | NR | NR | NR | NR | NR | NR | NR | NR | SIG 3.9 (2.3–6.6) | NR | NR | NR |  |
| **Foot-related Conditions** | | |  |  |  |  |  |  |  |  |  |  |  |  |  |  |
| **Foot infection** | |  |  |  |  |  |  |  |  |  |  |  |  |  |  |  |
| Foot infection | | NR | NR | NR | NR | NR | NR | SIG 1.91 (1.09–3.34) | NS 0.8 (0.6–1.2) | NR | NR | NR | NR | NR | NR |  |
| MDRB infection | | NR | NR | NR | NR | NR | NR | NR | NR | NR | NS 0.84 (0.64–1.11)*, 0.77 (0.59–1.01)**, 0.81 (0.59–1.10)*** | NR | NR | NR | NR |  |
| Gangrene | | NR | NR | NR | NR | NR | NR | NR | NS 0.9 (0.7–1.4) | NR | NR | NR | NR | NR | NR |  |
| Ulcer site | | NR | NR | NR | NR | NR | NR | NS 0.99 (0.60-1.66) | NR | NR | NR | NR | NR | NR | NR |  |
| Ulcer area | | NR | NR | NR | NR | NR | NR | NS 0.90 (0.58-1.40) | NR | NR | NR | NR | NR | NR | NR |  |
| Depth of ulcer | | NR | NR | NR | NR | NR | NR | NS 0.97 (0.55-1.69) | NR | NR | NR | NR | NR | NR | NR |  |
| Peripheral Neuropathy | | NR | NR | NR | NR | NR | NR | NR | NR | NR | NR | SIG 2.24 (1.35–3.71) | SIG 4.10 (1.87–8.97) | NR | NR |  |
| **Peripheral Arterial Disease** | |  |  |  |  |  |  |  |  |  |  |  |  |  |  |  |
| Peripheral Arterial Disease | | NR | NR | NR | NR | NR | NR | NR | NR | NR | NR | SIG 1.85 (1.10–3.13) | NR | NR | NR |  |
| Intermittent claudication | | NR | NR | NR | NR | NR | NR | NR | NR | NR | NR | SIG 2.77 (1.52–5.04) | NR | NR | NR |  |
| Ischaemia | | NR | NR | NR | NR | NR | NR | SIG 2.03 (1.31–3.14) | SIG 2.1(1.6–5.5) | NR | NR | NR | NR | NR | NR |  |
| History of peripheral re-vascularisation | | NR | NR | NR | NR | NR | NR | NR | NR | NR | NR | NR | SIG 7.96 (3.28–19.30) | NR | NR |  |
| Pulse pressure (5mm Hg increase) | | NR | NR | NR | NR | NR | NR | NR | NR | NR | NR | NS 1.07 (1.00–1.14) | NR | NR | NR |  |
| Absence of any foot pulse | | NR | NR | NR | NR | NR | NR | NR | NR | NR | NR | NR | SIG 2.46 (1.28–4.71) | NR | NR |  |
| **Management and service related** | | | |  |  |  |  |  |  |  |  |  |  |  |  |  |
| Insulin management | | NR | NR | NR | NR | SIG | NR | NR | NR | NR | NR | NR | SIG 3.36 (1.74–6.48) | NR | NR |  |
| Slow acting | |  |  |  |  | M: 3671.2 (3211.0–4131.4) F: 2357.5 (1984.0–2731.1) |  |  |  |  |  |  |  |  |  |  |
| Rapid acting | |  |  |  |  | M: 4587.7 (4311.4–4863.9) F: 3016.7 (2790.6–3242.8) |  |  |  |  |  |  |  |  |  |  |
| Oral antidiabetic drugs | | NR | NR | NR | NR | SIG | NR | NR | NR | NR | NR | NR | NR | NR | NR |  |
| Not secretagogue | |  |  |  |  | M: 1589.2 (1478.0–1700.5) F:1074.2 (975.6–1172.8) |  |  |  |  |  |  |  |  |  |  |
| Secretagogue | |  |  |  |  | M: 2294.4 (2018.5–2570.3) F: 1455.4 (1243.7–1667.1) |  |  |  |  |  |  |  |  |  |  |
| Metformin usage Vs not usage of patients with renal diseases | | NR | NR | NR | NR | NR | NR | NR | NR | NR | NR | NR | NR | NR | SIG |  |
|  | |  |  |  |  |  |  |  |  |  |  |  |  |  | 0.38 (0.30-0.48^)#^ |  |
|  | |  |  |  |  |  |  |  |  |  |  |  |  |  | 0.41(0.32-0.53^)##^ |  |
|  | |  |  |  |  |  |  |  |  |  |  |  |  |  | 0.35(0.27- 0.46^)###^ |  |
|  | |  |  |  |  |  |  |  |  |  |  |  |  |  | 0.33 (0.23–0.49^)  ####^ |  |
| **Recent professional foot care** | |  |  |  |  |  |  |  |  |  |  |  |  |  |  |  |
| Preulcerative foot care within 12 months of diagnosis | | NR | NR | NR | NR | NR | NR | NR | NR | NR | NR | NR | NR | SIG 0.88 (0.82–0.94) | NR |  |
| Podiatrist visits Vs no podiatrist visits | | NR | NR | SIG | NR | NR | NR | NR | NR | NR | NR | NR | NR | NR | NR |  |
| Commercial I insurance | |  |  | 0.73 (0.70- 0.76) |  |  |  |  |  |  |  |  |  |  |  |  |
| Medicare insurance | |  |  | 0.84 (0.81- 0.87) |  |  |  |  |  |  |  |  |  |  |  |  |
| Late referral | | NR | NR | NR | NR | NR | NR | NR | SIG 4.4 (2.6–11.1) | NR | NR | NR | NR | NR | NR |  |
| Multidisciplinary limb preservation program | | NR | NR | NR | SIG^ p<0.0001 | NR |  | NR | NR | NR | NR | NR | NR | NR | NR |  |
| Standard of care | |  |  |  | 1.50% |  |  |  |  |  |  |  |  |  |  |  |
| Multideciplinary care | |  |  |  | 1.10% |  |  |  |  |  |  |  |  |  |  |  |

SIG- Significant, NS- Not significant, NR- Not reported, M- Male, F- Female, T1- Type 1 diabetes, T2- Type 2 diabetes, $- Multivariable analysis was not performed in this study, ^ Confidence intervals not reported, +Adjusted for age, sex and race-centre, ++ Adjusted for age, sex, race-centre, SES, health insurance, smoking and alcohol consumption, +++Adjusted for age, sex, race-centre, SES, health insurance, smoking, alcohol consumption BMI, hypertension, chronic kidney disease, prevalent CHD status, HDL-cholesterol, triacylglycerol and LDL cholesterol, *Adjusted for age and gender, ** Adjusted for bone compromise, severe infection, and peripheral arterial disease, *** Adjusted for diabetes mellitus duration and HbA1C, # Adjusted for age, sex, and comorbidities ((hypertension, liver disease, dyslipidemia, cardiovascular disease, cerebrovascular disease, and previous diabetic foot event), ## Adjusted for age, sex, comorbidities, and initial laboratory findings (serum creatinine, glycated haemoglobin [HbA1c], and serum albumin), ### Adjusted for age, sex, comorbidities, initial laboratory findings, and medication usage (sulfonylurea, dipeptidyl peptidase-4 inhibitor, insulin, and renin–angiotensin system blocker), #### Propensity score matching (adjusted for age, sex, smoking status, dyslipidemia, coronary artery disease, cerebral vascular disease, liver disease and previous diabetic foot, initial estimated glomerular filtration rate, initial HbA1c, and medications). MDRB- Multi Drug Resistance bacteria, BMI- Body mass index, HDL- High density lipoproteins, LDL- Low density lipoproteins, HbA1c – glycated haemoglobin, eGFR- Estimated glomerular filtration rate, uACR- Urine albumin- creatinine ratio, CHD- coronary heart disease, ****Population from same study, however Hamilton et.al. had many more participants and explore different risk factors for hospitalisation

**Table S7:** Definitions for factors identified as risk factors by included studies

|  | Risk factor | Definition | Reference/ Citation |
| --- | --- | --- | --- |
|  | **Sociodemographic variables** |  |  |
|  | Sex (Males) | Not reported |  |
|  | Education status | Educational level for each individual was obtained by record linkage with the 2011 National Census and was classified into three levels: high (university or high school), medium (middle school), and low (primary school or no formal education). | Monge et al., 2020 |
|  | Height (1cm increase) | Not reported |  |
|  | Alcohol consumption (1stand/day) | Alcohol consumption (for a 1 standard drink/day increase)10 units per day | Baba et al., 2014 |
|  | **Biomedical measures** |  |  |
|  | HbA1c (for a 1.0% increase) | Not reported | Baba et al., 2014, Hamilton et al., 2021 |
|  |  |  |  |
|  | **Comorbidities** |  |  |
|  | Diabetes (vs no diabetes) | ≥1 HbA1c test ≥48 mmol/mol (6.5%); or ≥1 fasting plasma glucose tests ≥7.0 mmol/L (126 mg/dL); or ≥1 random plasma glucose test ≥11.1 mmol/L (200 mg/dL) recorded in either Royal Hobart Hospital Pathology or Diagnostic Services Pty Ltd (DSPL); or Hospital diagnostic code (primary or other) in the E10–E14 ranges recorded in either AP | Dinh et al., 2023 |
|  |  | Fasting glucose ≥7 mmol/l, non-fasting glucose ≥11.1 mmol/l, self-report of a diagnosis of diabetes by a physician, or use of glucose-lowering medication at study visit 1 | Fang et al., 2021 |
|  | Type 1 (vs Type 2) diabetes | The type of diabetes was retrieved from the Regional Diabetes Registry (RDR) of the Piedmont Region, Italy. | Monge et al., 2020 |
|  |  | Registrants were classified as having type 1 diabetes if they were assigned type 1 diabetes by the registering healthcare practitioner, and met any one of the following criteria: 1) there was less than a year between diagnosis of diabetes and their first prescription for insulin, 2) when date of diagnosis is missing, there was evidence of insulin use at registration on the NDSS and the registrant was <45 years of age of registration, or 3) for those with an age of diagnosis <30 years (or if missing a diagnosis date, registration at age <45 years) who registered on the NDSS prior to 2002, and whose insulin initiation date on the NDSS was missing, there had to be evidence of ongoing treatment with insulin early in the years we had data from the PBS available (from 2002). Additionally, type 1 status was assigned to registrants whose original assignment was type 2, but were <30 years old at diabetes onset and showed evidence of insulin use within a year, a pattern more consistent with type 1 diabetes. An additional requirement for assigning type 1 was evidence of ongoing treatment with insulin (≥2 prescriptions for insulin on the PBS), except when time to census/death was <2 years. Individuals satisfying none of these criteria were classified as having type 2 diabetes. Additionally, because type 2 diabetes is rarely diagnosed in children under 10 years of age, registrants who were classified as having type 2 diabetes diagnosed at <10 years of age were excluded from all analyses to decrease the likelihood of including people with type 1 diabetes. | Quigley et al., 2022 |
|  | Age at diabetes diagnosis | Not reported | Hamilton et al., 2021 |
|  | Cardiovascular disease (CVD) | Data on existing cardiovascular disease (CVD) were obtained by record linkage with the regional hospital discharge database, which contains data on all hospitalizations of Piedmont residents wherever they may have been hospitalized in Italy. All patients discharged from hospital in the previous 5 years with a primary or secondary diagnosis of coronary heart disease (ICD 9-CM: 410–414) or cerebrovascular disease (ICD 9-CM: 430–438) were defined as having CVD | Monge et al., 2020 |
|  | Cerebrovascular disease | Self-reported stroke and transient ischaemic attack were amalgamated with prior hospitalisations to define baseline cerebrovascular disease | Baba et al., 2014 |
|  | End stage renal failure | Dialysis was considered in the case of end-stage-renal-disease (ESRD) requiring renal replacement therapy. | Meloni et al., 2021 |
|  | Previous dialysis | Considered as affected from CKF all the patients who had a dialytic treatment in the previous 5 years | Monge et al., 2020 |
|  | Estimated glomerular filtration rate (eGFR) eGFR<60mL/min | The estimated glomerular filtration rate (eGFR) was calculated using the Chronic Kidney Disease Epidemiology Collaboration equation | Baba et al., 2014 |
|  | ln(uACR) (mg/mmol) * A 2.72-fold increase in uACR corresponds to an increase of 1 in ln(uACR) | Albuminuria assessed by early morning spot urinary albumin-to-creatinine ratio (uACR) measurement | Hamilton et al., 2021 |
|  | Retinopathy | Any grade of retinopathy, including maculopathy, was detected by direct and/or indirect ophthalmoscopy in one or both eyes and/or on more detailed assessment by an ophthalmologist. | Baba et al., 2014 |
|  | **Foot-related conditions** |  |  |
|  | Foot infection | Diagnosis of infection was defined according to IWGDF guidelines; A pathological state of the foot, caused by invasion and multiplication of microorganisms in host tissues accompanied by tissue destruction and/ or a host inflammatory response | Meloni et al., 2021 |
|  |  | Clinical signs of infection of either soft tissues or bone (as proposed by the Infectious Disease Society of America (IDSA) and IWGDF) | Ha Van et al., 2023 |
|  | MDRB infection | Lack of susceptibility to at least one agent in three or more classes of antimicrobials for each bacterial genus: Staphylococcus sp., Enterococcus sp., Enterobacteriaceae, Pseudomonas sp., and Acinetobacter sp. Innate resistances to some drugs were not considered for this definition | Yovera et al., 2024 |
|  | Gangrene | Diagnosis of infection was defined according to IWGDF guidelines: A condition that occurs when the body tissue dies because of insufficient blood supply, infection, or injury | Meloni et al., 2021 |
|  | Ulcer site | Midfoot or Hindfoot | Ha Van et al., 2023 |
|  | Ulcer area | Area, or the two maximum dimensions at right angles multiplied, Ulcer > 1 cm² | Ha Van et al., 2023 |
|  | Depth of ulcer | Ulcer deep‐reaching muscle, tendon, joint capsule or bone | Ha Van et al., 2023 |
|  | Peripheral Neuropathy | Score of >2/8 on the clinical portion of the Michigan Neuropathy Screening Instrument (MNSI) | Baba et al., 2014 |
|  |  | Score of >2/8 on the clinical portion of the Michigan Neuropathy Screening Instrument | Hamilton et al., 2021 |
|  |  | Neuropathy, or loss of protective sensation on the basis of examination using 10‐g nylon monofilaments | Ha Van et al., 2023 |
|  | Peripheral artery disease | Ankle brachial indices (ABI) were <= 0.90 on either leg or a diabetes-related amputation was present | Baba et al., 2014 |
|  | Intermittent claudication | Pain in the calves came on during walking, caused the patient to slow down or stop, and resolved with rest | Baba et al., 2014 |
|  | Ischaemia | Either no palpable distal pedal pulses, TcPO2 < 30mmhg and/or arterial stenosis/occlusions documented by ultra-sound duplex or computed tomography or MRI requiring lower limb revascularization. | Meloni et al., 2021 |
|  |  | Clinical evidence of reduced pedal blood flow; no pulse palpable with signs of poor perfusion (cold feet, skin discolouration, slower hair growth, swelling, cramping) with or without gangrene | Ha Van et al., 2023 |
|  | History of peripheral vascularisation |  | Hamilton et al., 2021 |
|  | Pulse pressure (5mm Hg increase) | Examination included a detailed bilateral assessment including palpation for the pedal pulses (dorsalis pedis and posterior tibial) | Baba et al., 2014 |
|  | Absence of any foot pulse |  |  |
|  | **Management and other** |  |  |
|  | Insulin management | Insulin (separately short acting or long acting). Patients prescribed both insulin and oral antidiabetics were included in the “insulin treatment” category. | Monge et al., 2020 |
|  |  | Patients were requested to bring all medications to each visit, and full details were recorded. | Hamilton et al., 2021 |
|  | Metformin usage | Medication usage was defined as a prescription for medication for longer than 90 days during the follow-up period. | Kwon et al., 2024 |
|  | Preulcerative outpatient foot care within 12 months of diagnosis | Received outpatient diabetic foot examination and/or foot care during the 12 months before ulceration using the following Current Procedural Terminology (CPT) codes: 11055, Trim skin lesion; 11056, Trim skin lesions, 2 to 4; 11057, Trim skin lesions over 4; 11719, Trim nail(s); 11720, Debridement of nail(s) by any method (s), 1 to 5; 11721, Debridement of nail(s), 6 or more; G0127, Trim nail(s); G0245, Initial evaluation or management of a diabetic patient with diabetic sensory neuropathy; G0246, Follow-up evaluation and management of a diabetic patient with diabetic sensory neuropathy; and G0247, Routine foot care by a physician of a diabetic patient with diabetic sensory neuropathy. | Tan et al., 2024 |
|  | Podiatrist visits | One or more visits to a podiatrist during the year prior to the index foot ulcer | Gibson et al., 2013 |
|  | Late referral to specialised diabetic foot services | According to the timing of referral, patients were divided in two groups: early referral (ER) and late referral (LR). Based on the fast-track pathway (FTP) recommendations, ER were considered patients who referred immediately after 2 weeks in the case of uncomplicated (superficial, not infected, not ischaemic ulcers) non-healing ulcers (reduction of ulcer size < 30% after 2 week of standard of care), within 4 days in the case of complicated ulcers (ischaemic, infected (mild/moderate), deep (involving soft tissue and/or bone), or any kind of ulcers in patients on dialysis or with heart failure) and within 24 h in the case of severely complicated ulcers (abscess, wet gangrene, necrotizing fasciitis or in the case of fever or clinical signs of sepsis). LR patients were considered when the specific timing of referral for each grade of ulcer’ severity was not respected. | Meloni et al., 2021 |
|  | Multidisciplinary limb preservation program | A podiatry-led multidisciplinary approach, emphasizes coordinated care and aids in the early detection of these risk factors, resulting in reduced LEA rates | Manji et al., 2024 |

HbA1c – glycated haemoglobin, uACR- Urinary albumin-to-creatinine ratio, IWGDF- International Working Group of Diabetes Foot, TcPO_2_ - Transcutaneous oxygen measurement, MRI- Magnetic resonance imaging
